# Supplementary material for: NANP targeting radiosensitizes glioblastoma through TNFR1 sialylation-driven mesenchymal shift
Source: Nat Commun. 2026 Mar 18;17:4130. doi: 10.1038/s41467-026-70853-x (PMC13149966; doi:10.1038/s41467-026-70853-x)
Supplement: Supplementary file 1 — Supplementary Information [file 41467_2026_70853_MOESM1_ESM.pdf]

Supplementary Information for

NANP Targeting Radiosensitizes Glioblastoma through TNFR1 Sialylation-Driven Mesenchymal Shift

Yingwen Ding[1,2,3*], Ze-Yan Zhang[1,2,3*], Ravesanker Ezhilarasan[2,3], Aram S. Modrek[2,3,4], Melanie Graciani[2,3], Jerome Karp[2,3], Graysen McManus[2,3], Ananya Jambhale[2,3], Erik P. Sulman[2,3,5#]

[1]School of Basic Medical Sciences, Institute of Biomedical Innovation, The MOE Basic Research and Innovation Center for the Targeted Therapeutics of Solid Tumors, Provincial Key Laboratory of Tumor Biology, Jiangxi Medical College, Nanchang University, China.
[2]Department of Radiation Oncology, New York University (NYU) Grossman School of Medicine, New York, NY, USA.
[3]Brain and Spine Tumor Center, Laura and Isaac Perlmutter Cancer Center, NYU Langone Health, New York, NY, USA.
[4]Department of Radiation Oncology, Keck School of Medicine of University of Southern California (USC), Los Angeles, CA, USA.
[5]Department of Radiation Oncology, Duke University School of Medicine, Durham, NC, USA.

[*] These authors contribute equally to this work.
[#]Correspondence to erik.sulman@duke.edu (E.P.S.)

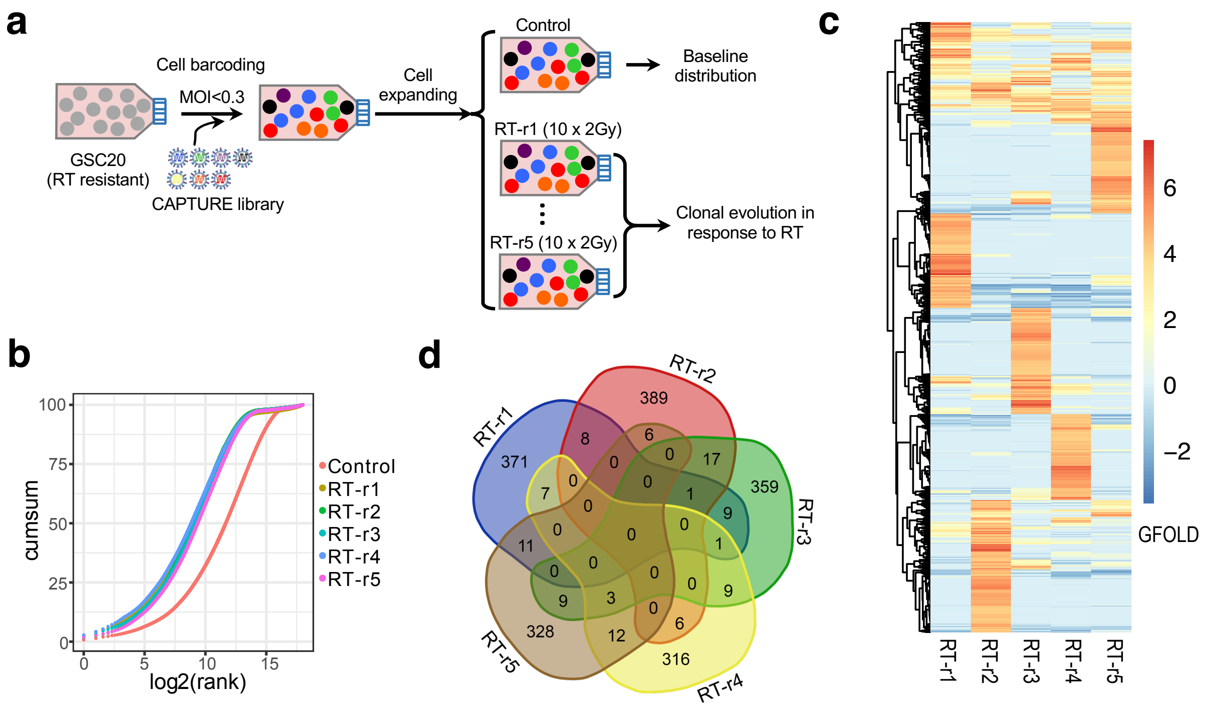

Supplementary Fig. 1: Clonal evolution following radiation.
(**a**) Schematic outline of the experimental design for clonal tracking using DNA barcoding approach. Created in PowerPoint with elements created in BioRender. Zhang, Z. (2025) https://BioRender.com/gnecstk (**b**) Cumulative plot of barcode percentages from each experiment replicate. (**c**) Heatmap of enriched barcodes (GFOLD log2 fold change > 4) from all replicates compared with baseline control group. (**d**) A Venn diagram showing the overlap of barcodes from (**c**) across the replicates.

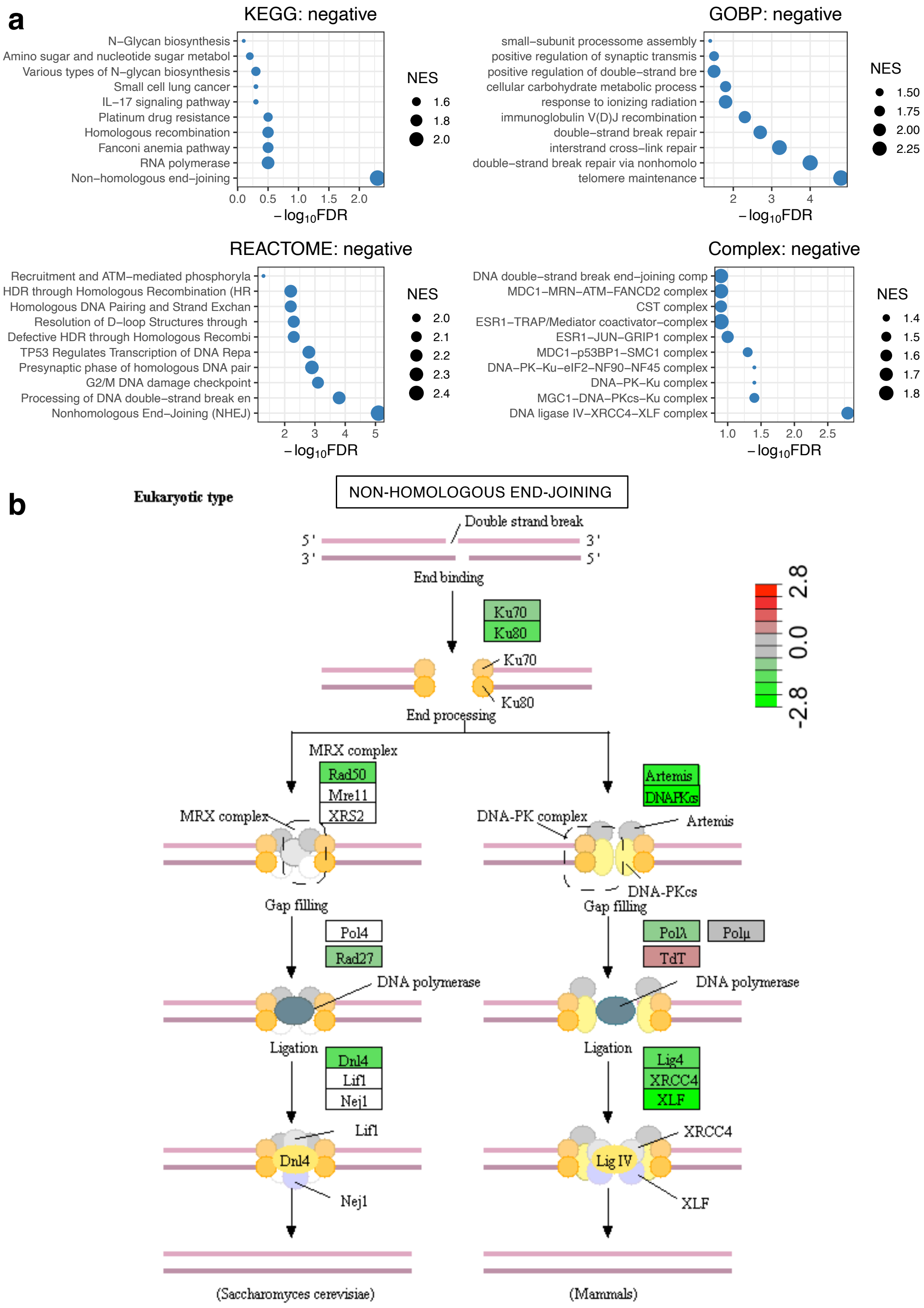

Supplementary Fig. 2: Enrichment analysis of CRISPR screening.
(**a**) KEGG, Gene Ontology Biological Process (GOBP), Reactome and Complex analysis of the negative selected genes from the CRISPR screening using MAGECKFlute. (**b**) KEGG pathway network showing the top enrichment pathway, NHEJ, with log2 fold changes highlighted by color. Created with the clusterProfiler[1] R package.

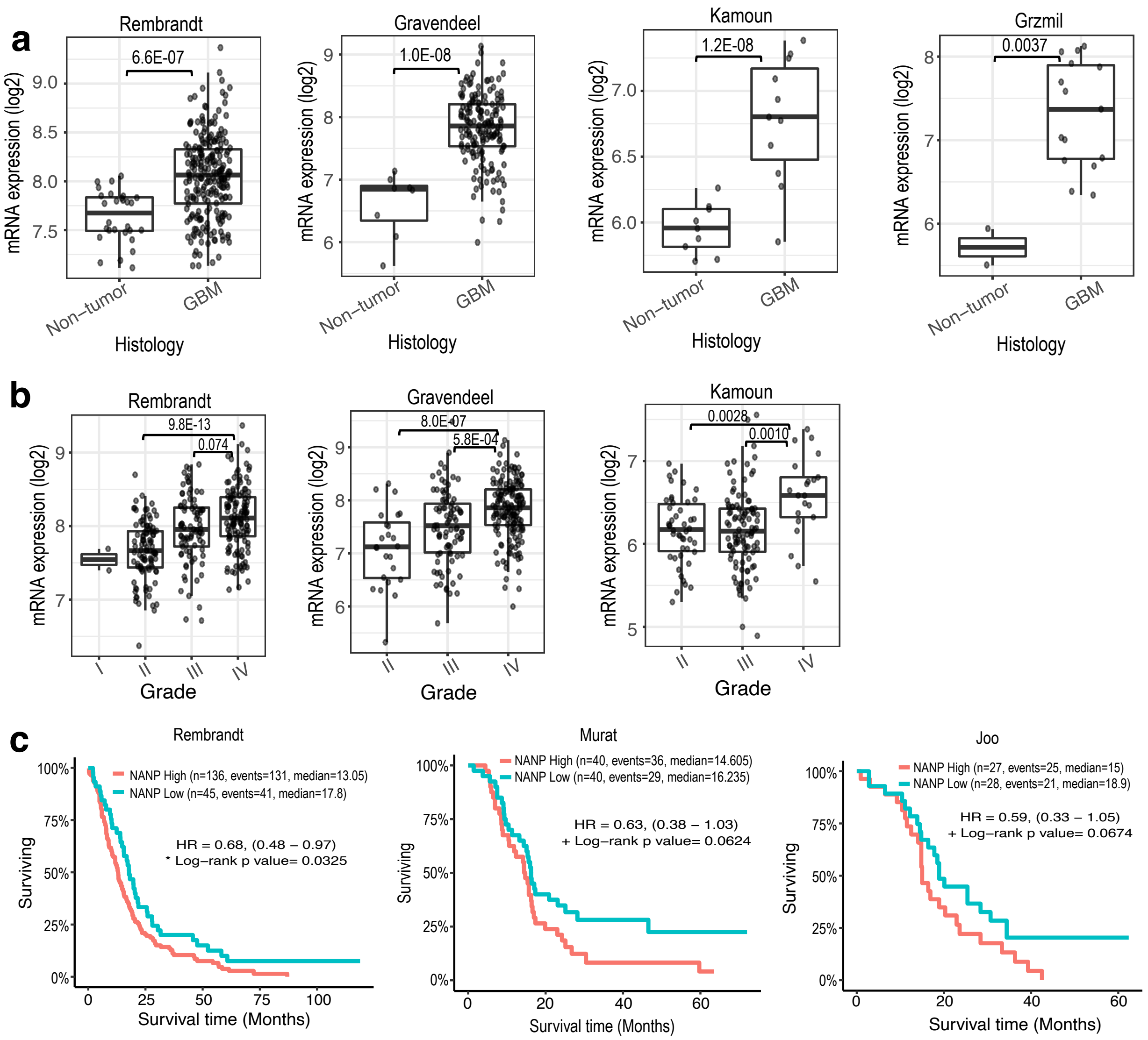

Supplementary Fig. 3: NANP expression in public patient cohorts.

(**a**) Boxplot showing the expression of *NANP* in non-tumor and GBM tissue from public patient cohorts as indicated. Unpaired t-test, two-tailed *p*-values are shown. (Rembrandt[2]: $n = 28$ for non-tumor, $n = 219$ for GBM; Gravendeel[3]: $n = 8$ for non-tumor, $n = 159$ for GBM; Kamoun[4]: $n = 9$ for non-tumor, $n = 11$ for GBM; Grzmil[5]: $n = 2$ for non-tumor, $n = 15$ for GBM) (**b**)Boxplot showing the expression of *NANP* in gliomas over grades from public patient cohorts as indicated. Unpaired t-test, two-tailed *p*-values were shown. (Rembrandt: $n = 2$ for grade I, $n = 98$ for grade II, $n = 85$ for grade III, $n = 130$ for grade IV; Gravendeel: $n = 24$ for grade II, $n = 85$ for grade III, $n = 159$ for grade IV; Kamoun: $n = 46$ for grade II, $n = 102$ for grade III, $n = 201$ for grade IV) Box plots (**a**-**b**) show the median (center line), IQR (box: 25th-75th percentiles), and whiskers extending to the minimum/maximum values within 1.5×IQR. (**c**) Kaplan-Meier survival analysis for overall survival between patients with high and low expression of *NANP* in the indicated patient cohorts. *$p<0.05$; + indicates $0.05 < p < 0.1$.

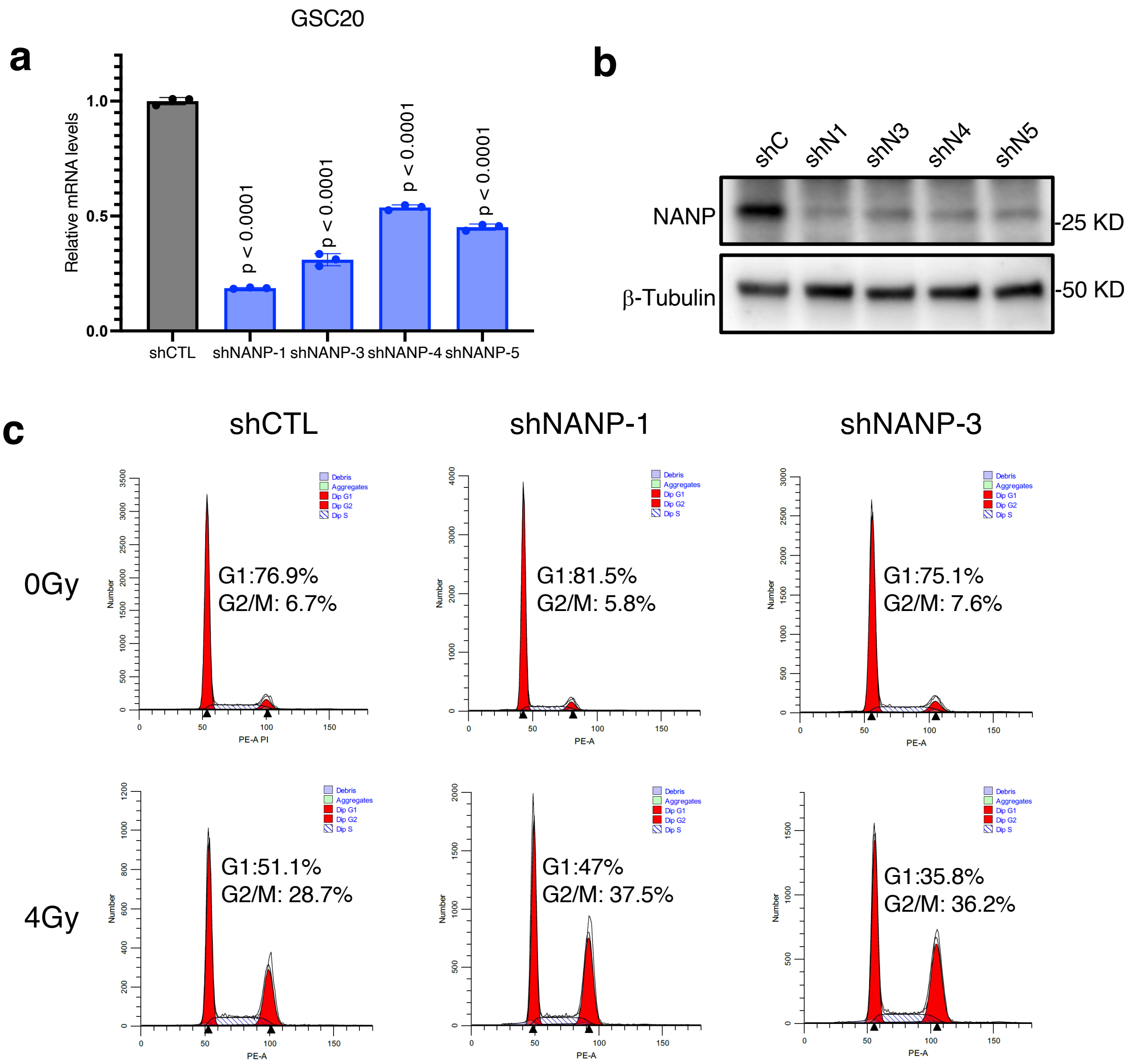

Supplementary Fig. 4: NANP knockdown efficiency validation and its effect on RT-induced cell cycle arrest in GSC20.

(**a**-**b**) Barplot showing knockdown efficiency of shRNAs targeting NANP (shNANP-1/3/4/5) versus control shRNA (shCTL) in GSC20 measured by qPCR (**a**) and immunoblot (**b**). 18S rRNA was used as loading control for qPCR and β-tubulin was used as loading control for immunoblot. Data are means ± SD. *$p < 0.05$, two-sided unpaired t-test used (n = 3 biological replicates). (**c**) Cell cycle analysis of GSC20 without or NANP silencing at 24h after radiation (0 Gy or 4 Gy). Similar results were obtained from three independent replicates.

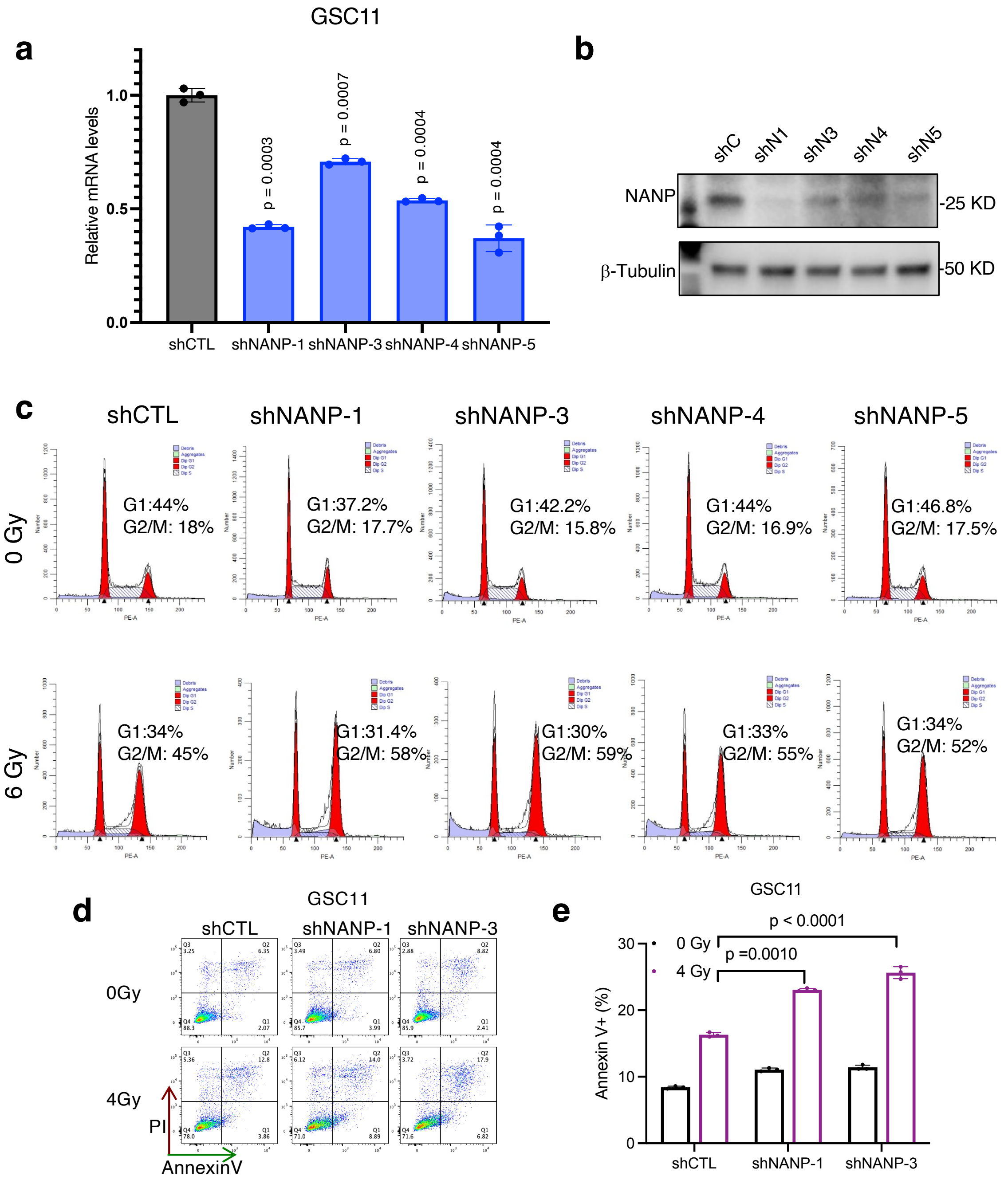

Supplementary Fig. 5: NANP knockdown efficiency validation and its effect on RT-induced cell cycle arrest in GSC11.

(**a**-**b**) Barplot showing knockdown efficiency of shRNAs targeting NANP (shNANP-1/3/4/5) vs. control (shCTL) in GSC11 by qPCR (**a**) or immunoblot (**b**). 18S rRNA was used as loading control for qPCR and β-tubulin was used as loading control for immunoblot. Data are means ± SD; two-sided, unpaired t-test used (*n* = 3 biological replicates). (**c**) Cell cycle analysis of GSC11 without or NANP silencing at 24h after radiation (0 Gy or 6 Gy). Similar results were obtained from three independent replicates for shNANP-1 and shNANP-3. (**d**-**e**) Apoptosis analysis of GSC11 (**d**-**e**) after 48 h of radiation using Annexin V-FITC-PI staining. Data are means ± SD; unpaired t-test used (*n* = 3 biological replicates). FITC, fluorescein isothiocyanate; PI, propidium iodide.

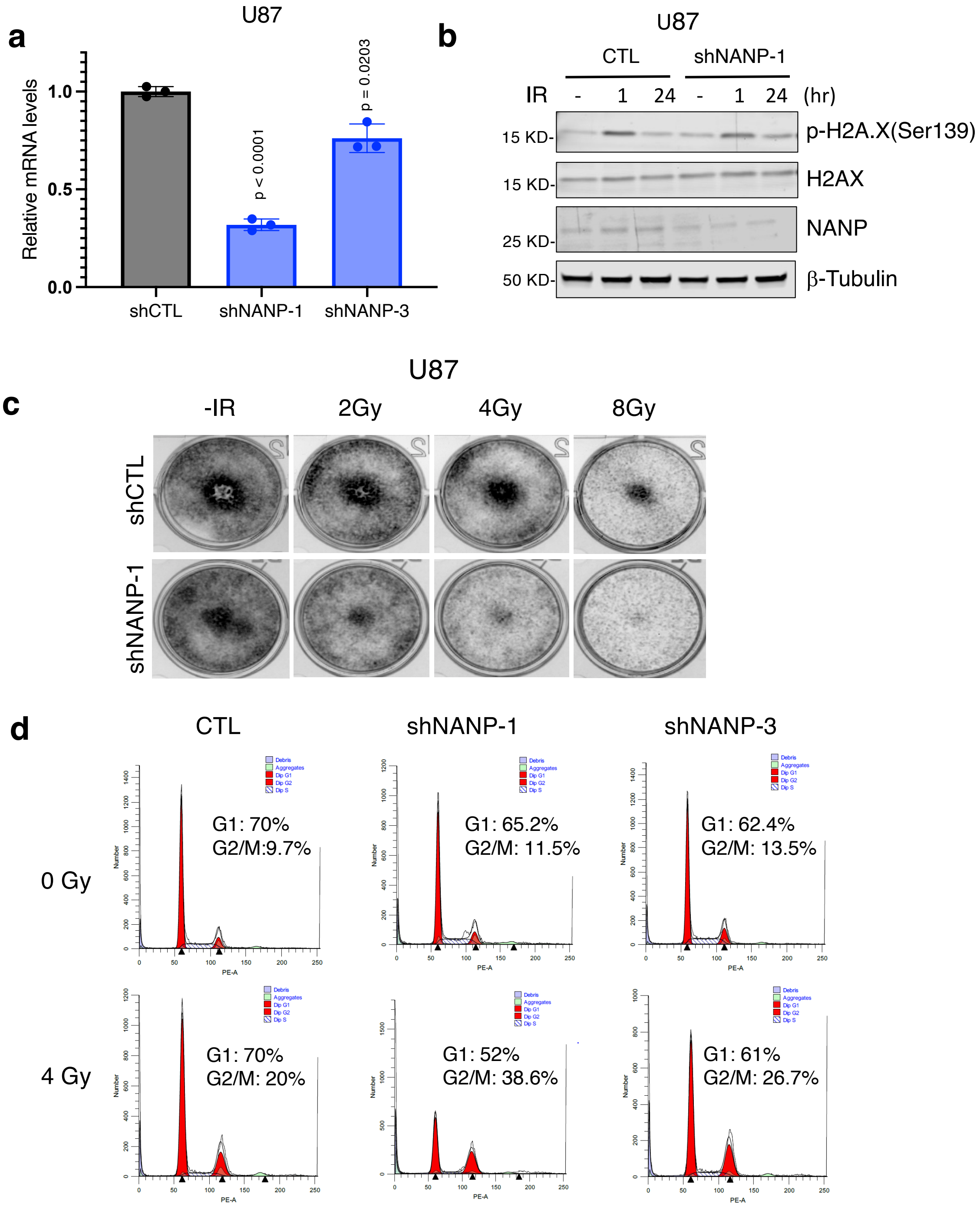

Supplementary Fig. 6: NANP knockdown efficiency validation and its effect on clonogenic assay, RT-induced γH2AX and cell cycle arrest in U87.

(**a**-**b**) Barplot showing knockdown efficiency of shRNAs targeting NANP (shNANP-1/3) vs. control (shCTL) in U87 by qPCR (**a**) or immunoblot (**b**). 18S rRNA was used as loading control for qPCR and β-tubulin was used as loading control for immunoblot. Data are means ± SD; two-sided, unpaired t-test used (n = 3 biological replicates). (**c**) Representative images of clonogenic assay of U87 with or without NANP silencing in response to RT. (**d**) Cell cycle analysis of U87 without or with NANP silencing at 24h after radiation (0 Gy or 4 Gy). For the immunoblots, colony formation, and cell cycle experiments, similar results were obtained in three independent replicates.

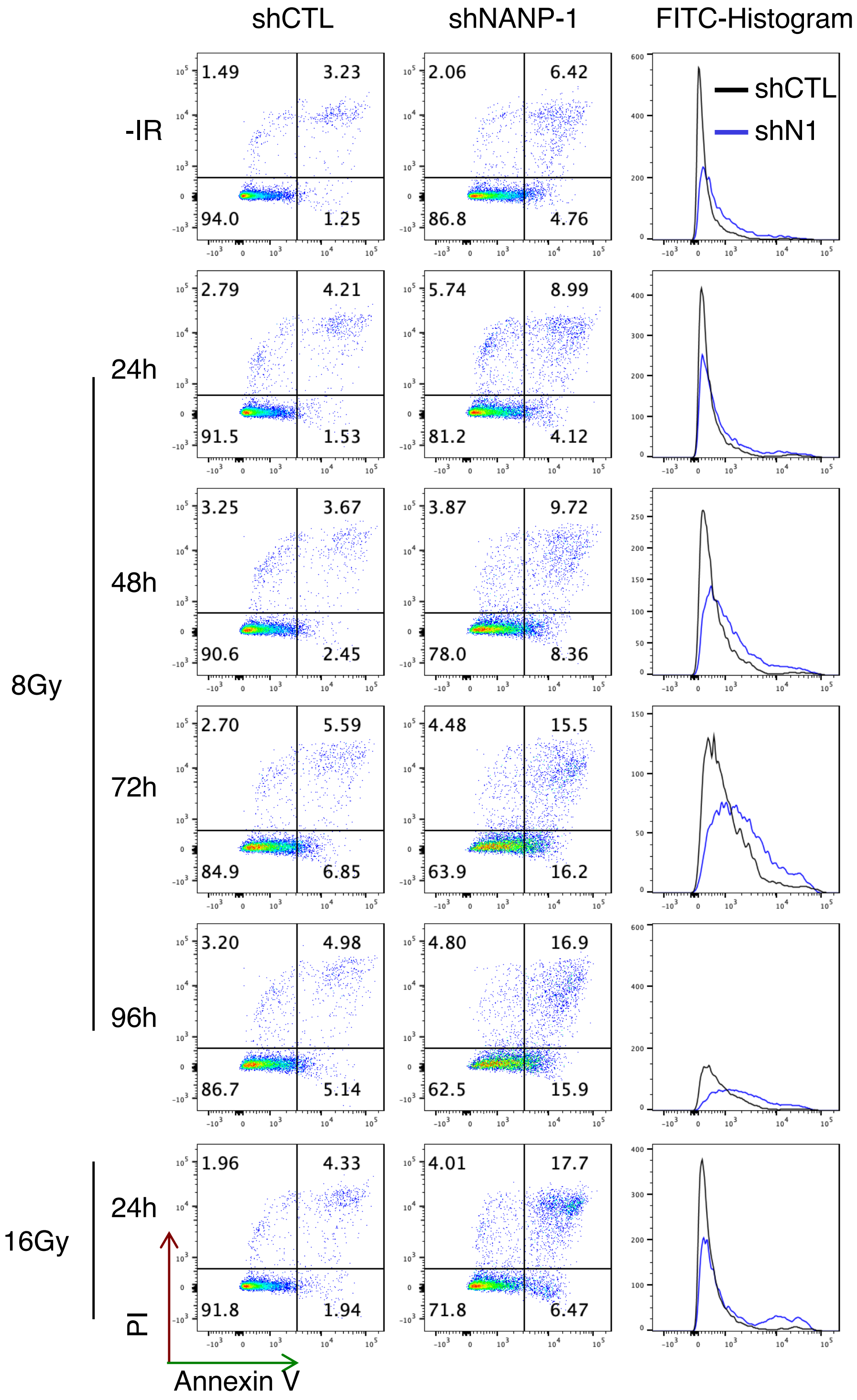

Supplementary Fig. 7: Time-course apoptosis analysis of shCTL and shNANP-1 GSC20 in response to RT. Scatter plots showing Annexin V-FITC and PI staining of indicated cells with indicated treatments. Parallel histogram showing FITC intensity versus cell count. Black line is for shCTL group; blue line is for shNANP-1 group. Similar results were obtained from two independent replicates.

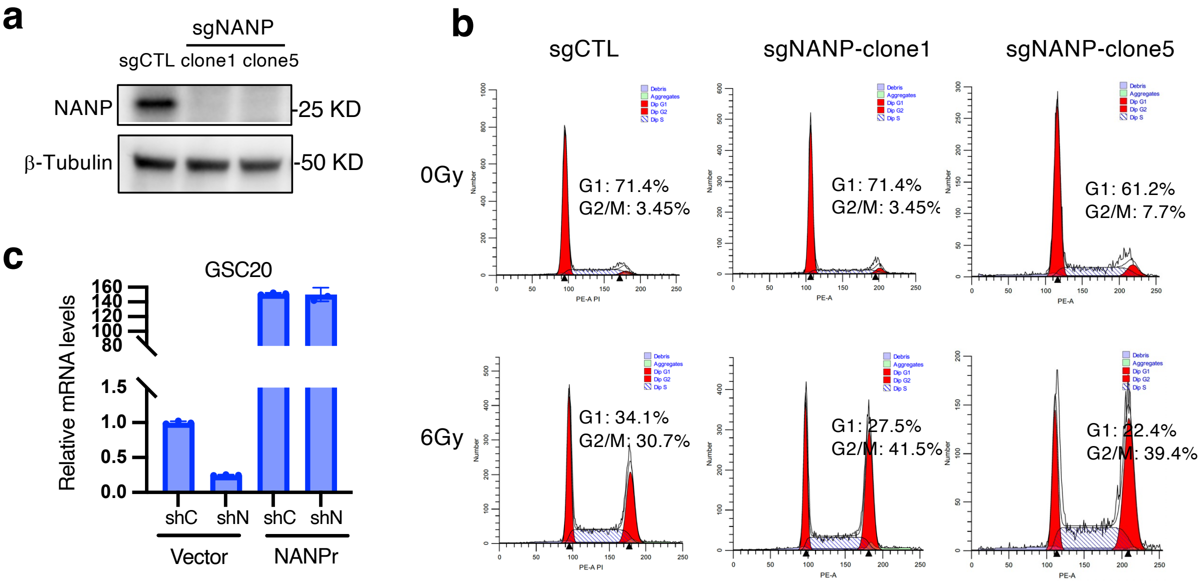

Supplementary Fig. 8 (**a**) Immunoblot results confirming the NANP knock-out. β-tubulin was used as loading control. (**b**) Cell cycle analysis of GSC20 without or with NANP knock-out at 24h after radiation (0 Gy or 6 Gy). Similar results were obtained from three independent replicates. (**c**) Real-time qPCR results confirming the rescue expression of *NANP* in NANP knock-down (shN) cells. Data are means ± SD, n = 3 technical replicates. Similar results of NANP knock-down or over-expression were obtained from different approaches in independent experiments.

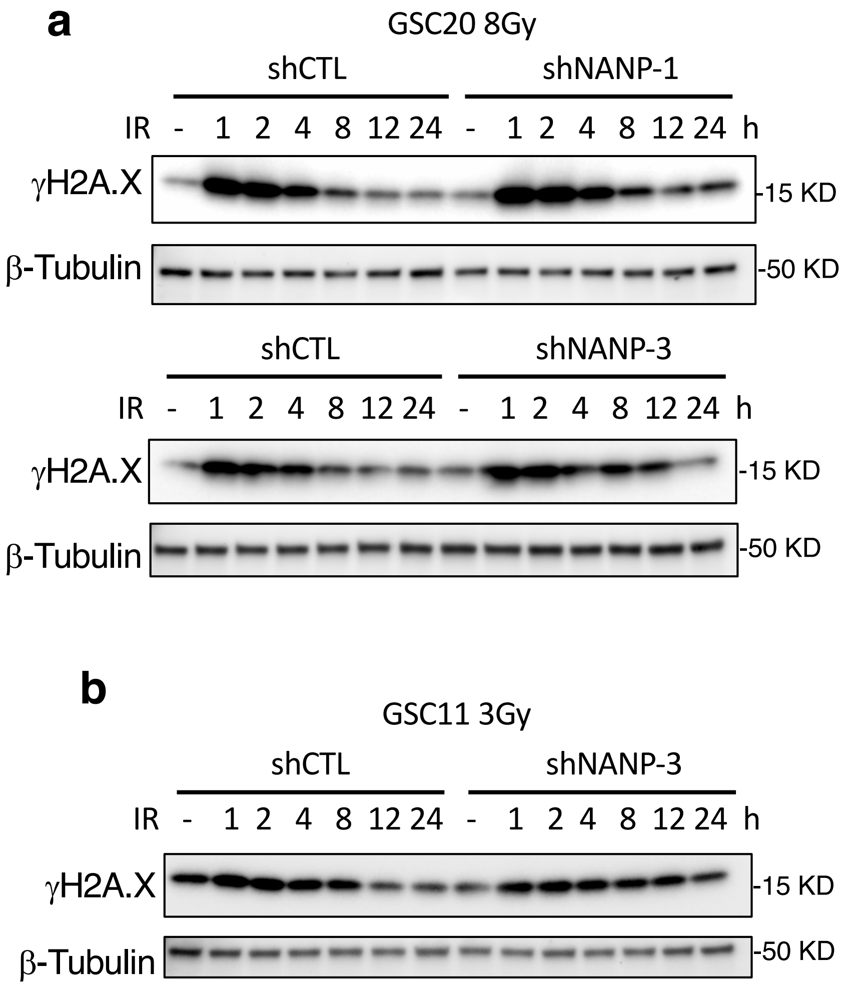

Supplementary Fig. 9: Time-course γH2AX levels analysis of shCTL and shNANP in GSC20 and GSC11 in response to RT. (**a**-**b**) Immunoblot results showing the dynamic of γH2AX levels in control (shCTL) or NANP silencing (shNANP-1 and/or shNANP-3) GSC20 (**a**) and GSC11 (**b**) harvested at the indicated time points. β-tubulin was used as loading control. Similar results of were obtained from three independent experiments.

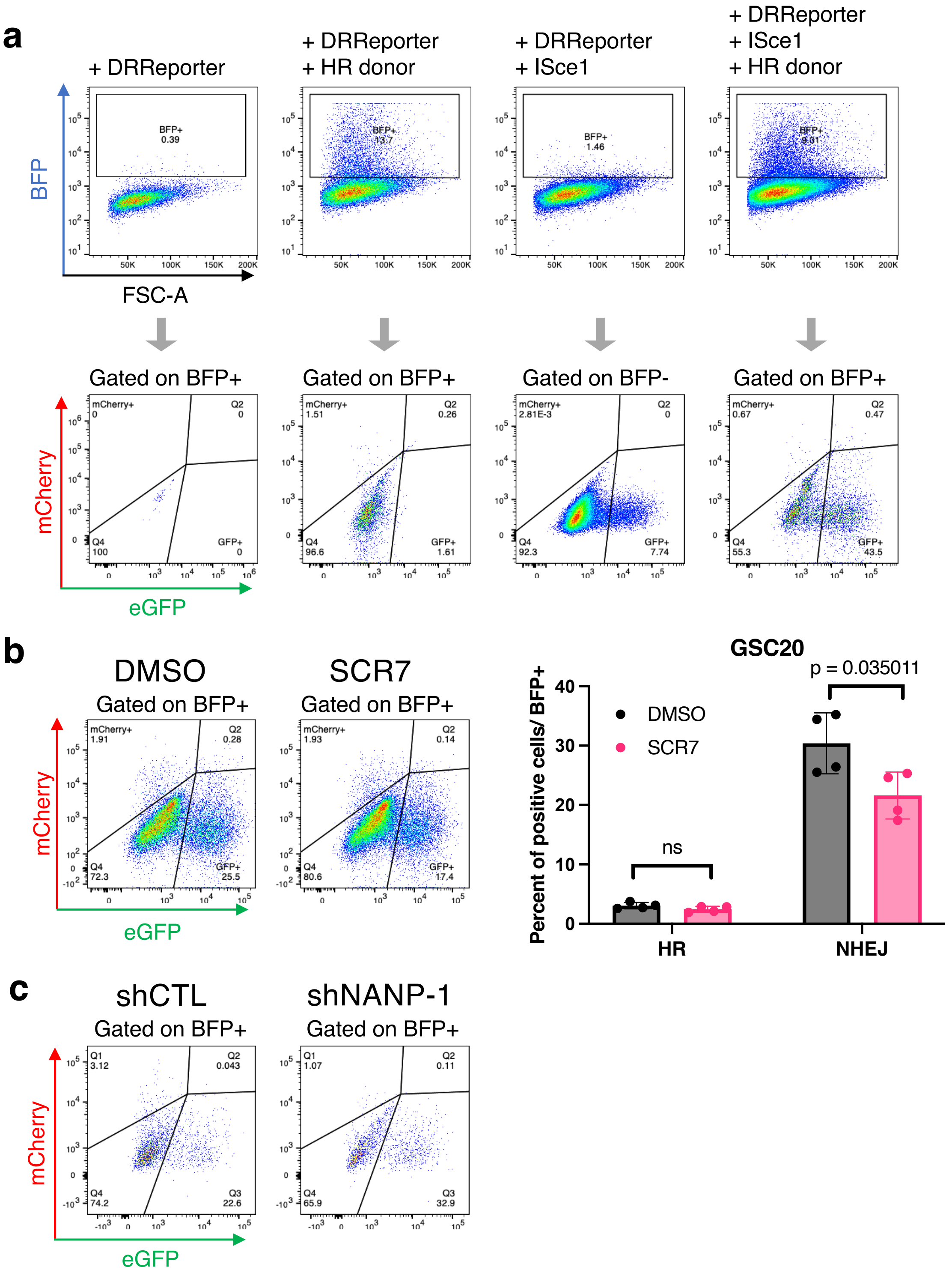

Supplementary Fig. 10: HR-NHEJ reporter assay.

(**a**) Scatter plots showing quality control of the reporter system. (**b**) Validation of the reporter system using a Lig4 inhibitor, SCR7. DMSO was vehicle control. Scatter plots showing the representative results, and the barplot showing the quantification results. Data are means±SD; two-sided, unpaired t-test used (n = 4 biological replicates). (**c**) Scatter plots showing the representative gating results of Figure 4j-k. Similar results were obtained from three independent replicates.

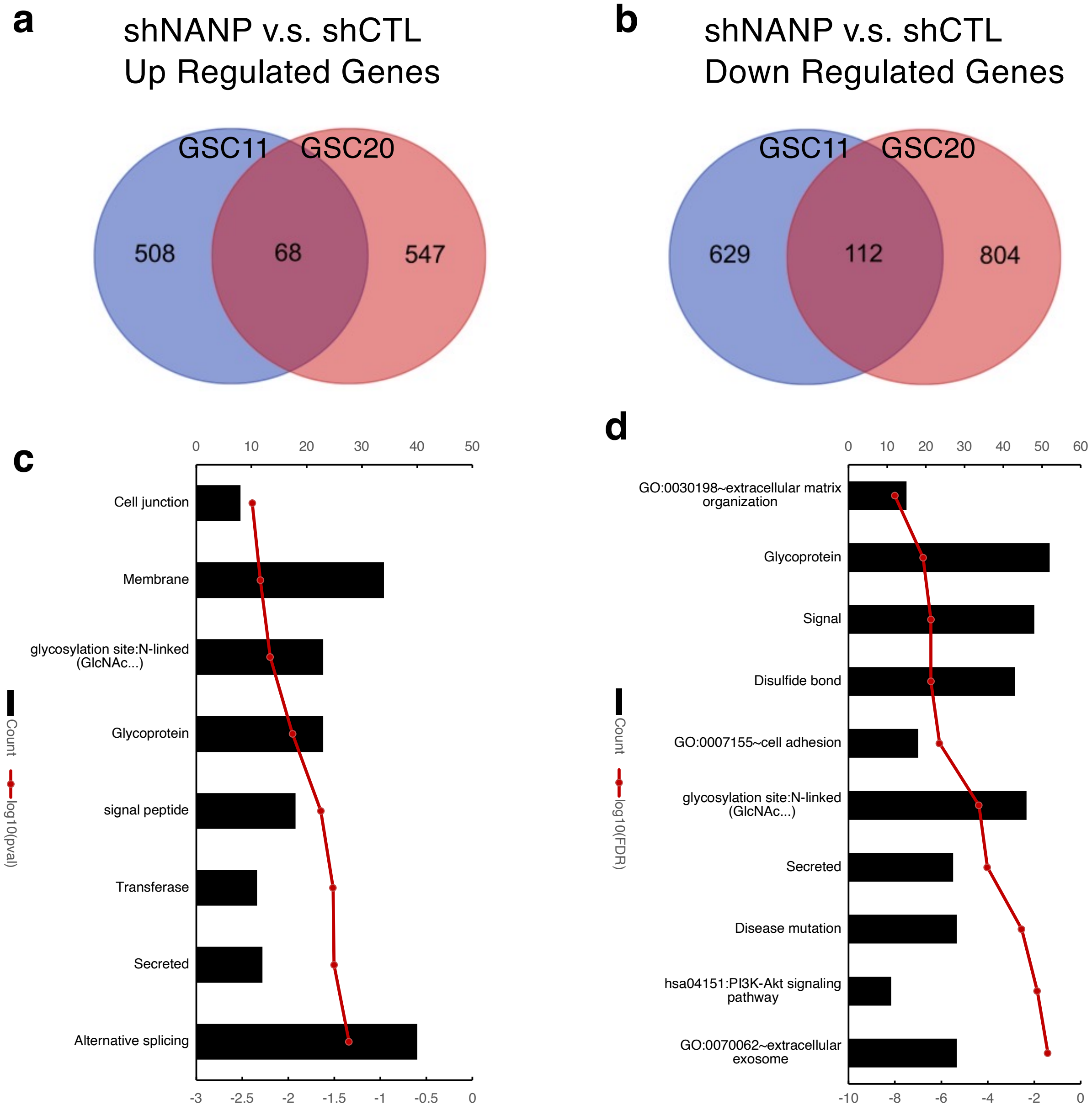

Supplementary Fig. 11: Common up- or down-regulated genes upon NANP suppression in GSC20 and GSC11 and overrepresenting pathways of these genes.

(**a-b**) Venn diagrams showing the overlapping of up-regulated (**a**) and down-regulated (**b**) genes upon NANP silencing in GSC20 and GSC11. (**c-d**) Barplots showing the top enriched terms of the common up-regulated (**c**) and down-regulated (**d**) genes shared by GSC20 and GSC11 upon NANP knockdown by DAVID webtool. The top x-axis represents the gene count, and the bottom x-axis represents the log10(FDR).

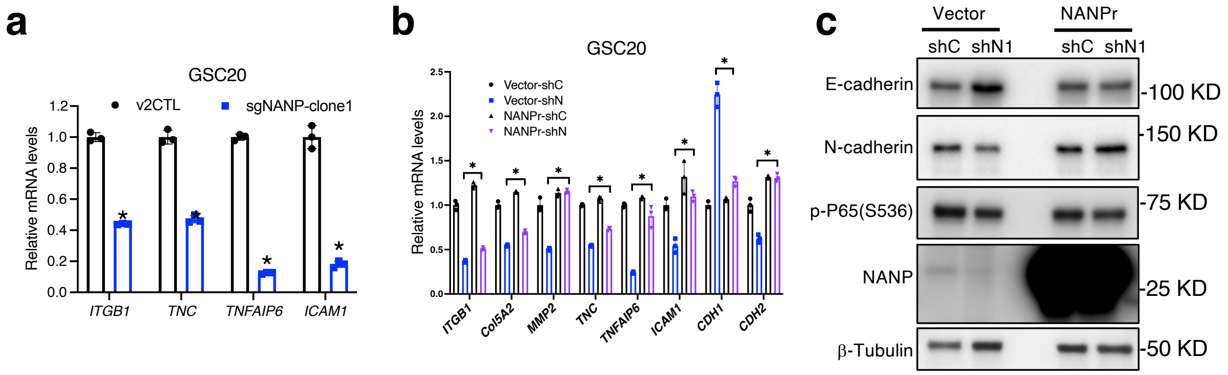

Supplementary Fig. 12

(**a**) Real-time qPCR results showing the NF-κB/MES genes expression of control(sgCTL) and NANP knock-out (sgNANP-clone1) cells. (**b**-**c**) Real-time qPCR (**b**) and immunoblot (**c**) results showing the NF-κB/MES genes expression of control (shC) or NANP knock-down(shN1) cells with shRNA-resistant NANP (NANPr) or vector expression. Data are means±SD; * $p < 0.05$ (exact p-values are provided in the Source Data file); two-sided, unpaired t-test used (n = 3 biological replicates). Similar results for the immunoblot experiment were obtained from three independent replicates.

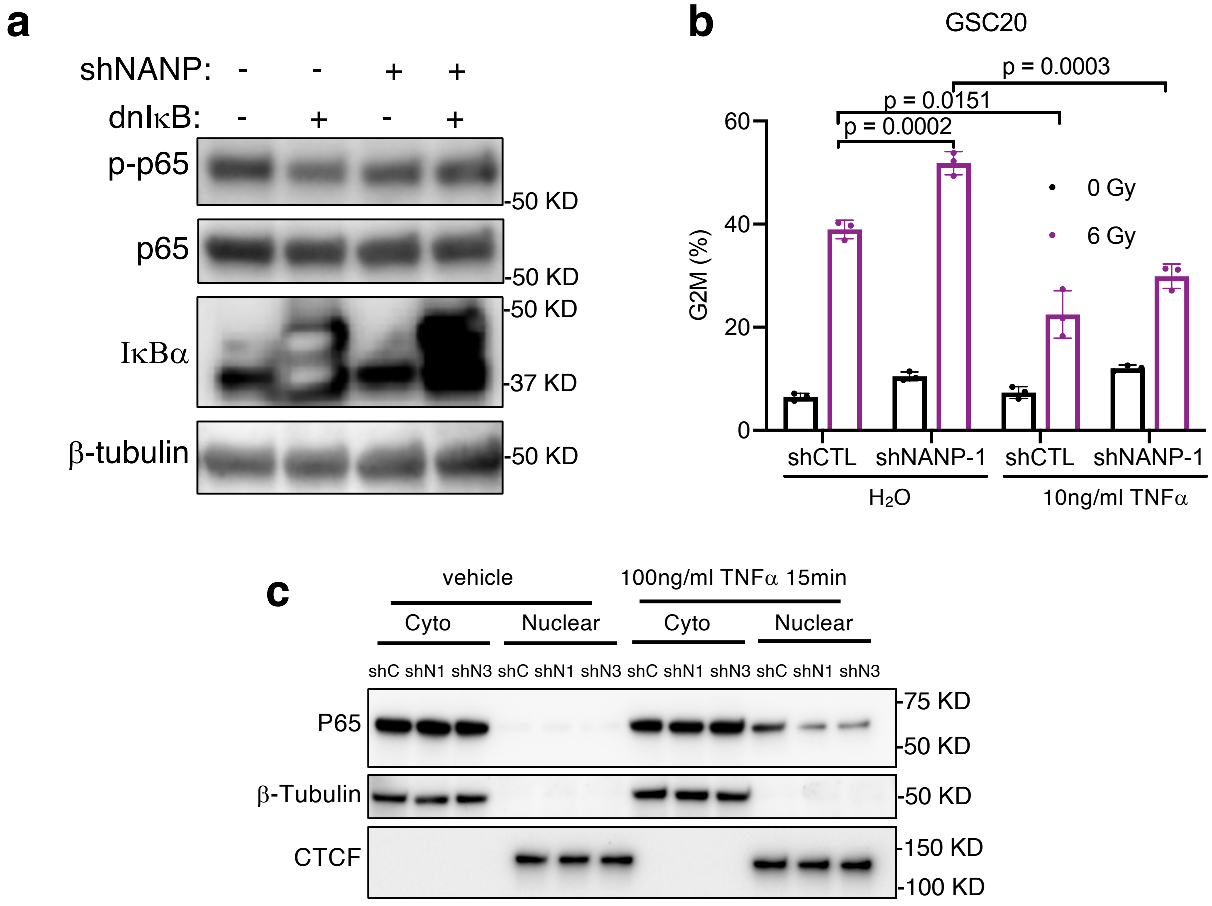

Supplementary Fig. 13

(**a**) Western blot analysis of p-p65, p65, IκBα in control and NANP silencing GSC20 in the condition of IκB inhibition dominant-negative IκB(dnIκB) overexpression or control (Vector). The β-tubulin was used as loading control. (**b**) Barplot showing the quantification results of Figure 6i. Data are means±SD; $^{\$}p < 0.05$ shCTL with TNFa compared with shCTL with water; $^{\#}p < 0.05$ shNANP with TNFα compared with shNANP with water. (Unpaired t-test used, $n = 3$ independent experiments). (**c**) GSC20 cells with indicated groups were fractionated into cytoplasmic and nuclear fractions. Immunoblot was performed to detected p65. CTCF was used as the nuclear marker and β-tubulin was used as cytoplasm marker. Similar results were obtained from three independent replicates.

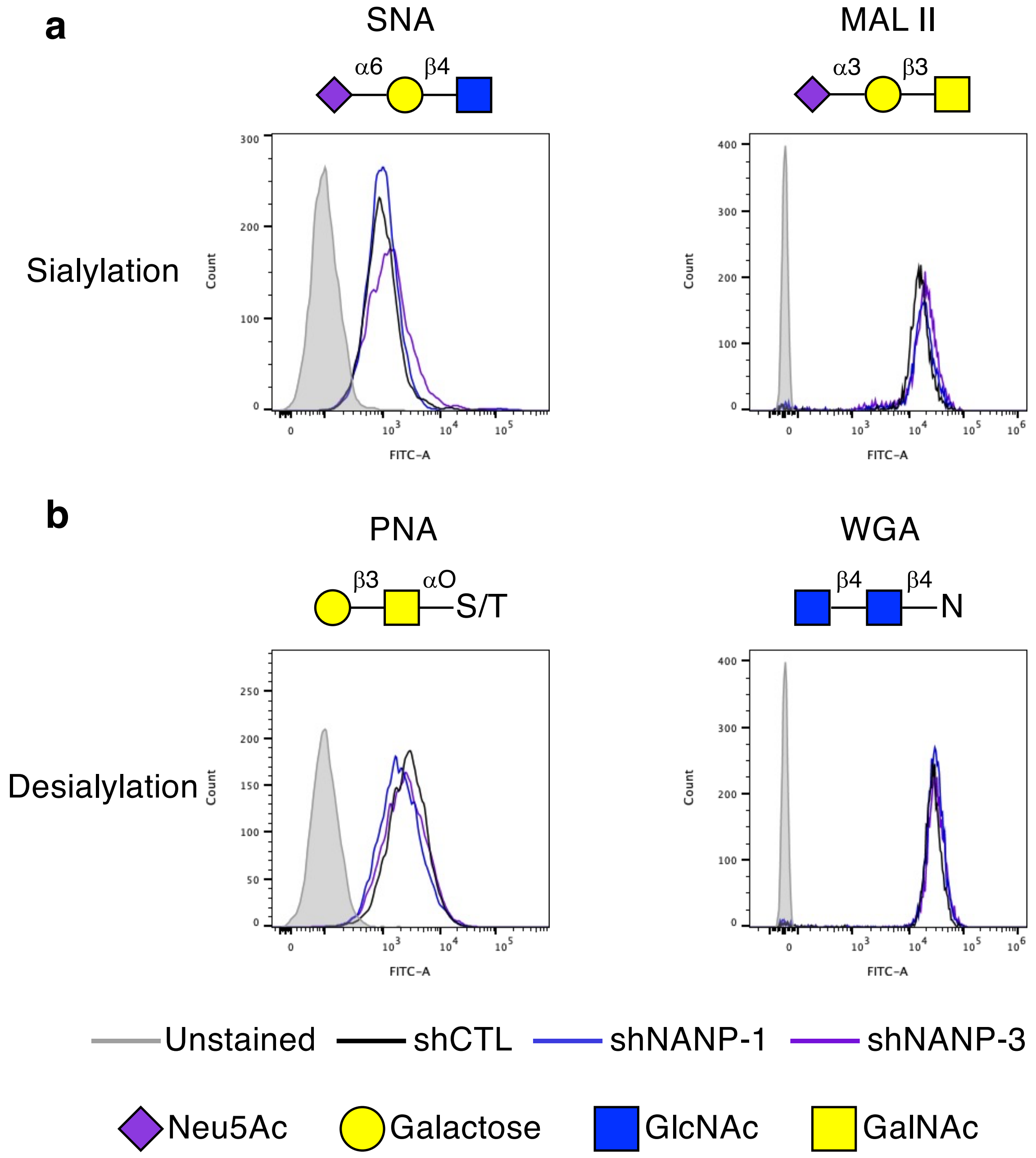

Supplementary Fig. 14: Cell surface sialylation analysis in control and NANP silencing GSC20.

(a) Flow cytometry analysis of SNA (*Sambucus nigra agglutinin*, α2,6-linked sialic acid) and MAL-II (*Maackia amurensis lectin II,* α2,3-linked sialic acid) binding in control and NANP knock down GSC20. (**b**) Flow cytometry analysis of PNA (*Peanut agglutinin*, β1,3-linked galactose) and WGA (*Wheat germ agglutinin*, β1,4-linked GlcNAc) binding in control and NANP knock down GSC20. Histogram show fluorescence intensity versus cell count. Gray peaks represent unstained negative control, black line is for shCTL group, blue line is for shNANP-1 group, purple line is for shNANP-3 group. Negative controls were treated equally as other samples, except omitting staining with the corresponding lectin. Similar results were obtained from two independent replicates.

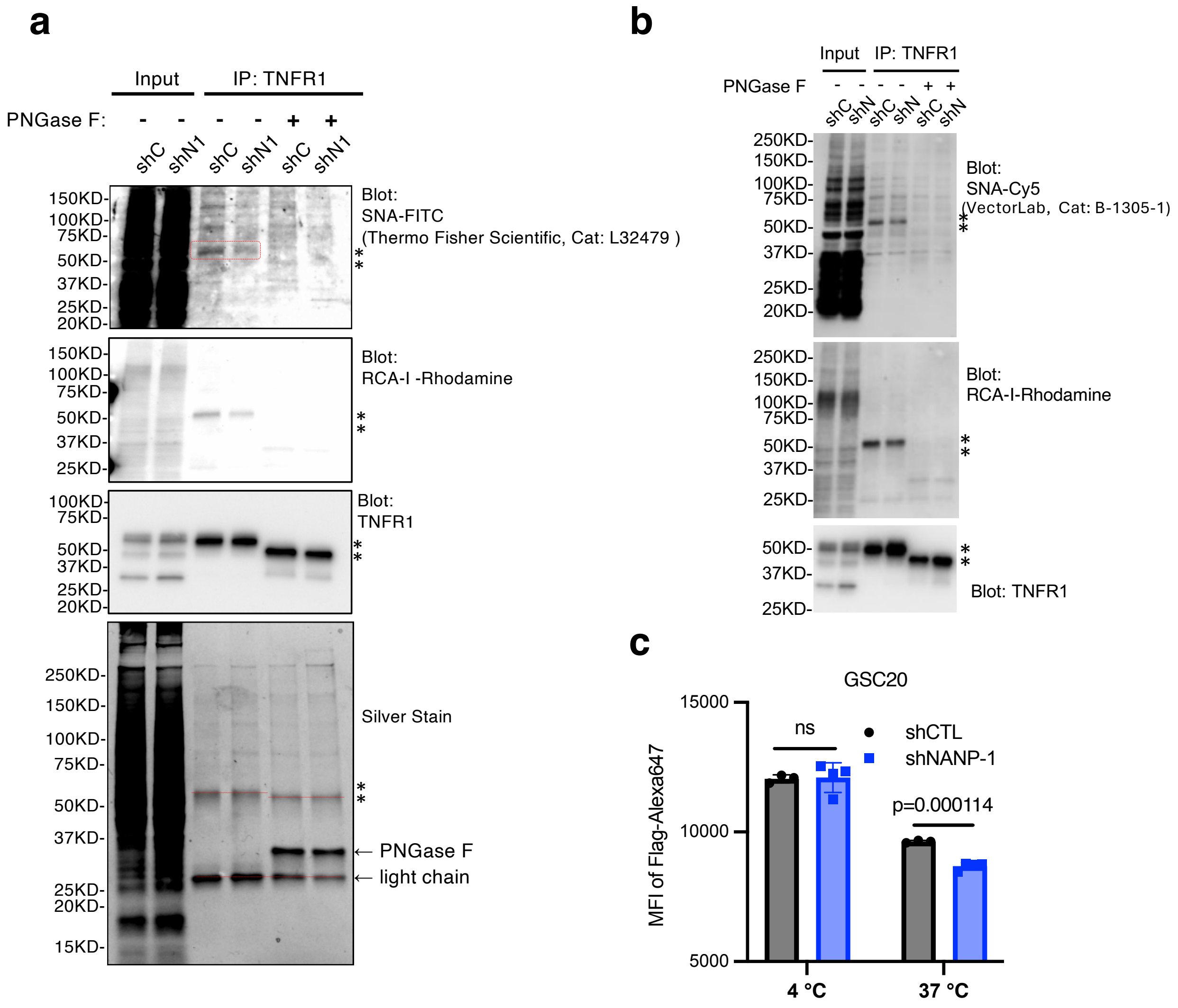

Supplementary Fig. 15

(**a**) Immunoprecipitation of endogenous TNFR1 followed with PNGase F digestion. TNFR1's α2,6-linked N-sialylation was detected by *Sambucus nigra agglutinin* (SNA-FITC) blotting and underlying galactose was detected by *Ricinus Communis Agglutinin I* (RCA I, RCA120) blot. Silver stain was used to check for the IP purity. PNGase F was used to confirm the sialyation bands. (**b**) Immunoprecipitation of endogenous TNFR1 followed with PNGase F digestion. TNFR1's α2,6-linked N-sialylation was detected by SNA-Cy5. * Indicates the molecular weight for TNFR1 with or without PNGase F digestion. (**a**) and (**b**) are independent replicates of the same experiment, and at least one more replicate with similar results was obtained. (**c**) Barplot showing the quantification results of Figure 6f. Data are means ± SD; two-tailed, unpaired t-test used, n = 3 biological replicates for shCTL and n = 4 biological replicates for shNANP-1.

Gating strategies for FACS data

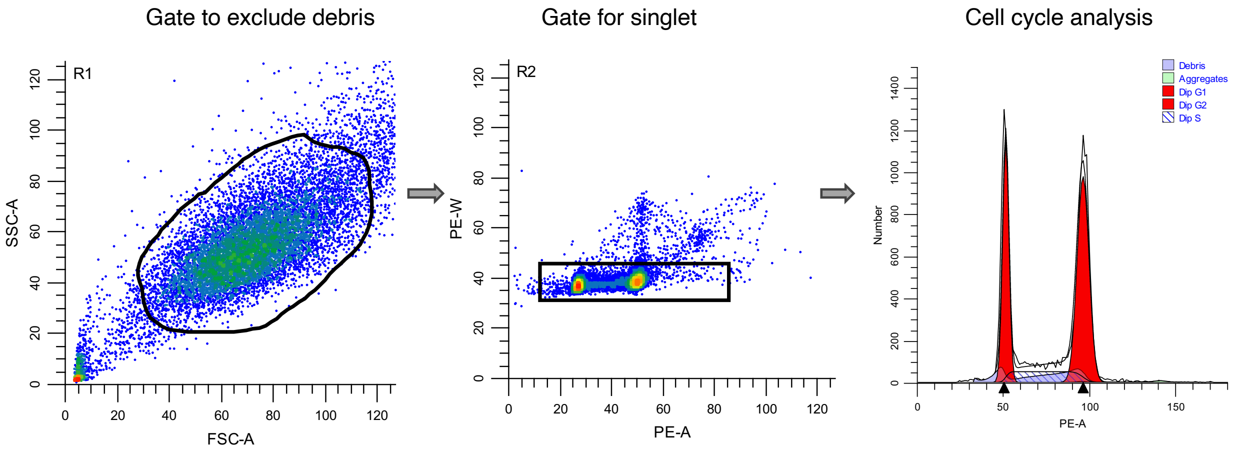

Supplementary Fig. 16: Gating strategies for PI staining cell-cycle analysis.
(Related to Figures 3c, 6i, Supplementary Fig. 4b, 5c, 6d and 8b.)

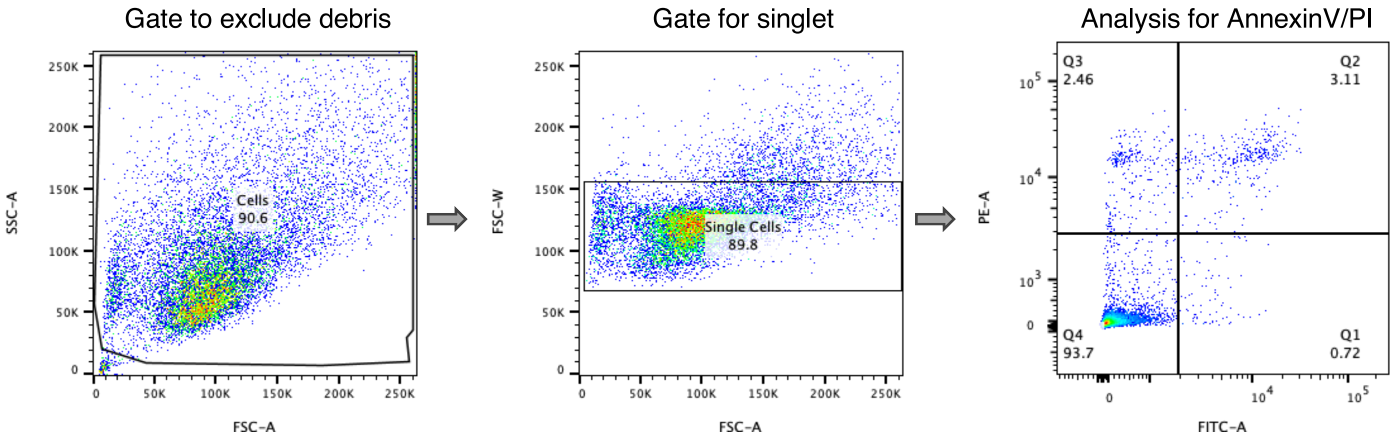

Supplementary Fig. 17: Gating strategies for AnnexinV/PI staining apoptosis analysis. (Related to Figure 3d, Supplementary Fig. 5d and 7.)

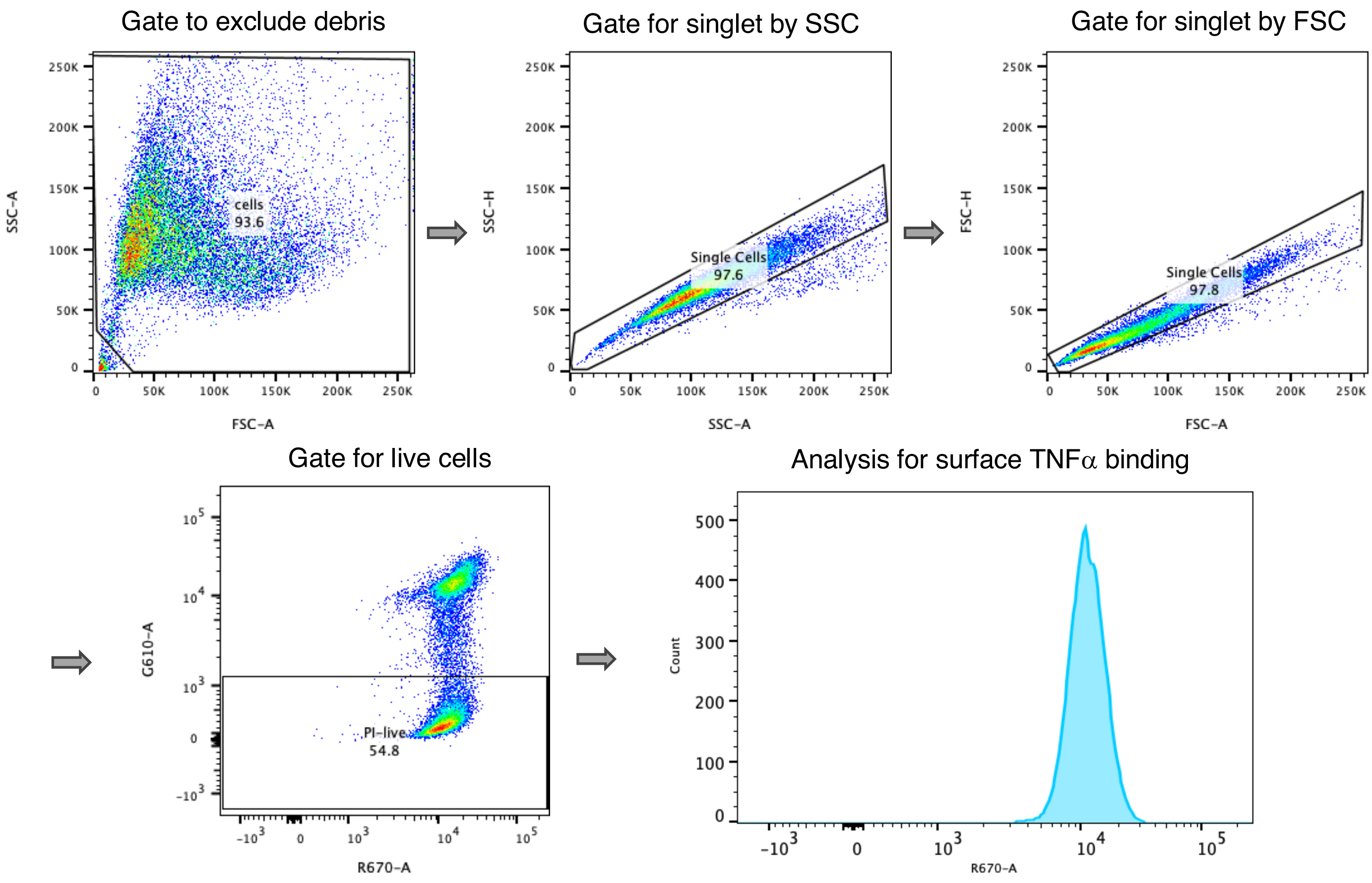

Supplementary Fig. 18: Gating strategies for TNFR1 analysis.
(Related to Figure 6f)

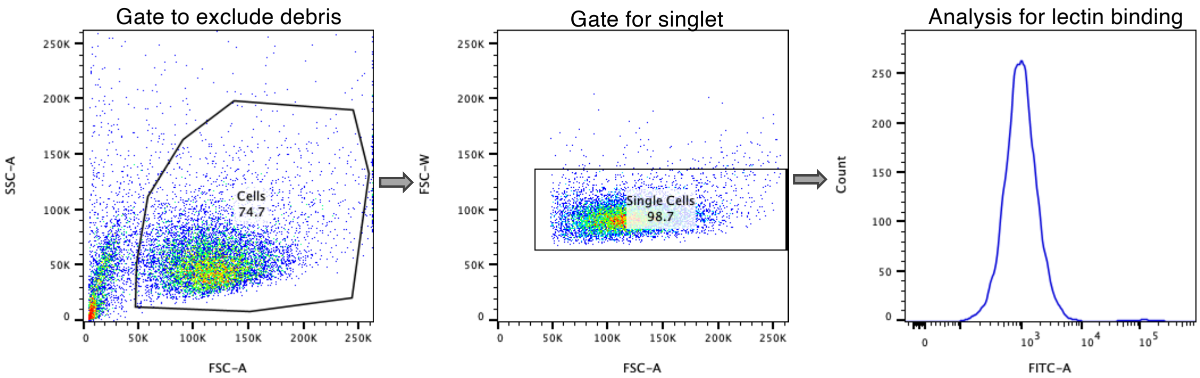

Supplementary Fig. 19: Gating strategies cell surface sialylation analysis. (Related to Supplementary Fig. 14)

References

1 Yu, G., Wang, L. G., Han, Y. & He, Q. Y. clusterProfiler: an R package for comparing biological themes among gene clusters. *OMICS* **16**, 284-287 (2012). https://doi.org:10.1089/omi.2011.0118

2 Madhavan, S. *et al.* Rembrandt: helping personalized medicine become a reality through integrative translational research. *Mol Cancer Res* **7**, 157-167 (2009). https://doi.org:10.1158/1541-7786.MCR-08-0435

3 Gravendeel, L. A. *et al.* Intrinsic gene expression profiles of gliomas are a better predictor of survival than histology. *Cancer Res* **69**, 9065-9072 (2009). https://doi.org:10.1158/0008-5472.CAN-09-2307

4 Kamoun, A. *et al.* Integrated multi-omics analysis of oligodendroglial tumours identifies three subgroups of 1p/19q co-deleted gliomas. *Nat Commun* **7**, 11263 (2016). https://doi.org:10.1038/ncomms11263

5 Grzmil, M. *et al.* MAP kinase-interacting kinase 1 regulates SMAD2-dependent TGF-beta signaling pathway in human glioblastoma. *Cancer Res* **71**, 2392-2402 (2011). https://doi.org:10.1158/0008-5472.CAN-10-3112
