# Supplementary material for: NANP targeting radiosensitizes glioblastoma through TNFR1 sialylation-driven mesenchymal shift
Source: Nat Commun. 2026 Mar 18;17:4130. doi: 10.1038/s41467-026-70853-x (PMC13149966; doi:10.1038/s41467-026-70853-x)
Supplement: Supplementary file 2 — Description of Additional Supplementary Files [file 41467_2026_70853_MOESM2_ESM.pdf]

Description of Additional Supplementary Files

Supplementary Data 1
Read per million(RPM) results of GS20 clonal evolution analysis in response to radiation treatment (RT). (relative to supplenmentary Fig.1)

Supplementary Data 2
CRISPR screening results (omit common essential genes) from MAGeCKFlute analysis.

Supplementary Data 3
A list of NANP correlated genes in TCGA GBM samples with $p<0.05$ by Pearson correlation analysis.

Supplementary Data 4
A list of differential expression genes (shNANP v.s. shCTL) in GSC20 and GSC11 from DESeq2 analysis.

Supplementary Data 5
A list of primer pairs for reverse transcription quantitative -polymerase chain reaction (RT-qPCR).
