# Supplementary material for: NANP targeting radiosensitizes glioblastoma through TNFR1 sialylation-driven mesenchymal shift
Source: Nat Commun. 2026 Mar 18;17:4130. doi: 10.1038/s41467-026-70853-x (PMC13149966; doi:10.1038/s41467-026-70853-x)
Supplement: Supplementary file 8 — Reporting Summary [file 41467_2026_70853_MOESM8_ESM.pdf]

nature portfolio

Corresponding author(s): Erik P. Sulman

Last updated by author(s): Feb 5, 2026

Reporting Summary

Nature Portfolio wishes to improve the reproducibility of the work that we publish. This form provides structure for consistency and transparency in reporting. For further information on Nature Portfolio policies, see our Editorial Policies and the Editorial Policy Checklist.

Statistics

For all statistical analyses, confirm that the following items are present in the figure legend, table legend, main text, or Methods section.

n/a	Confirmed	
☐	☒	The exact sample size (*n*) for each experimental group/condition, given as a discrete number and unit of measurement
☐	☒	A statement on whether measurements were taken from distinct samples or whether the same sample was measured repeatedly
☐	☒	The statistical test(s) used AND whether they are one- or two-sided<br>*Only common tests should be described solely by name; describe more complex techniques in the Methods section.*
☐	☒	A description of all covariates tested
☐	☒	A description of any assumptions or corrections, such as tests of normality and adjustment for multiple comparisons
☐	☒	A full description of the statistical parameters including central tendency (e.g. means) or other basic estimates (e.g. regression coefficient) AND variation (e.g. standard deviation) or associated estimates of uncertainty (e.g. confidence intervals)
☐	☒	For null hypothesis testing, the test statistic (e.g. *F*, *t*, *r*) with confidence intervals, effect sizes, degrees of freedom and *P* value noted<br>*Give P values as exact values whenever suitable.*
☒	☐	For Bayesian analysis, information on the choice of priors and Markov chain Monte Carlo settings
☒	☐	For hierarchical and complex designs, identification of the appropriate level for tests and full reporting of outcomes
☐	☒	Estimates of effect sizes (e.g. Cohen's *d*, Pearson's *r*), indicating how they were calculated

Our web collection on statistics for biologists contains articles on many of the points above.

Software and code

Policy information about availability of computer code

Data collection	All data was collected with commercially available and/or previously published methods. Please see Materials and Methods and Supplementary Information for details.
Data analysis	MAGeCK 0.5.8, MAGeCKFlute 3.19, R studio, R version 4.4.1, gfold 1.1.4, Trimmomatic 0.36, STAR 2.5, subread 1.6.3, DESeq2 1.18, GSEA 4.3.2, ssgsea.GBM.classification R package, GraphPad Prism 10, OpenComet v1.3.1, ImageJ bundled with Java 8, FlowJo 10

For manuscripts utilizing custom algorithms or software that are central to the research but not yet described in published literature, software must be made available to editors and reviewers. We strongly encourage code deposition in a community repository (e.g. GitHub). See the Nature Portfolio guidelines for submitting code & software for further information.

Data

Policy information about availability of data

All manuscripts must include a data availability statement. This statement should provide the following information, where applicable:

- Accession codes, unique identifiers, or web links for publicly available datasets
- A description of any restrictions on data availability
- For clinical datasets or third party data, please ensure that the statement adheres to our policy

All transcriptomics data generated in this study has been deposited in the public repository NCBI Gene Expression Omnibus(GEO; https://www.hcbi.nlm.nih.gov/geo/) under accession GSE274135.Raw data for the genome-wide CRISPR screen and clonal evolution analysis are available from Zenodo (https://doi.org/10.5281/zenodo.18466897). Other data that support the findings of this research are available within the paper and its Supplementary Information. Source Data are

provided with this paper.

Research involving human participants, their data, or biological material

Policy information about studies with human participants or human data. See also policy information about sex, gender (identity/presentation), and sexual orientation and race, ethnicity and racism.

Reporting on sex and gender	n/a
Reporting on race, ethnicity, or other socially relevant groupings	n/a
Population characteristics	n/a
Recruitment	n/a
Ethics oversight	n/a

Note that full information on the approval of the study protocol must also be provided in the manuscript.

Field-specific reporting

Please select the one below that is the best fit for your research. If you are not sure, read the appropriate sections before making your selection.

[x] Life sciences [ ] Behavioural & social sciences [ ] Ecological, evolutionary & environmental sciences

For a reference copy of the document with all sections, see nature.com/documents/nr-reporting-summary-flat.pdf

Life sciences study design

All studies must disclose on these points even when the disclosure is negative.

Sample size	For mouse experiment, assuming the survival time is 60 vs 100 days between two groups (15-day standard deviation), to have at least 80% power (α=0.05) to detect the extent of deviation in survival using a one-sided Wilcoxon rank-sum test, the sample size should be at least 5 for each experimental condition.
Data exclusions	No data were excluded.
Replication	Experiments were performed with independent biological replication and technical replicates.The findings were successfully reproduced.
Randomization	The mice were randomly allocated into experiment groups.
Blinding	The lab technician who took responsibility for monitoring the mouse survival was blinded to the group allocations.

Reporting for specific materials, systems and methods

We require information from authors about some types of materials, experimental systems and methods used in many studies. Here, indicate whether each material, system or method listed is relevant to your study. If you are not sure if a list item applies to your research, read the appropriate section before selecting a response.

Materials & experimental systems

n/a	Involved in the study	
[ ]	[x]	Antibodies
[ ]	[x]	Eukaryotic cell lines
[x]	[ ]	Palaeontology and archaeology
[ ]	[x]	Animals and other organisms
[x]	[ ]	Clinical data
[x]	[ ]	Dual use research of concern
[x]	[ ]	Plants

Methods

n/a	Involved in the study	
[x]	[ ]	ChIP-seq
[ ]	[x]	Flow cytometry
[x]	[ ]	MRI-based neuroimaging

Antibodies

Antibodies used	phospho-H2AX (Ser139) (MilliporeSigma, 05-636), NANP (SantaCruz, sc-374637), E-cadherin (CST, 3195), N-cadherin (CST, 13116), Slug (CST, 9585), ß-catenin(CST, 8480), pospho-IKKα/ß Ser176/180(CST, 2697), IKKα(CST, 11930), IKKß(CST, 8943), phospho-p65 (CST, 3033),p65 (CST, 8242), IκBα (CST, 1814), anti-tubulin (Biolegend, 801202), horse radish peroxidase (HRP)-linked anti-rabbit IgG (CST, 7074), anti-mouse IgG (CST, 7076) , anti-TNC (CST, 93029) , anti-CTCF (CST,2899),anti-PDPN(CST, 9047), anti-Flag(Alexa Fluor™ Plus 647, Invitrogen, 701629RP647)
Validation	All primary antibodies used in the study are commercially available and validation data for the species reactivity against human antigens and their respective application are noted on the manufacturer's website.

Eukaryotic cell lines

Policy information about cell lines and Sex and Gender in Research

Cell line source(s)	Patient-derived glioblastoma stem-like cells (GSC20, GSC11) were previously generated in-house(PMID:23993863) and were maintained in neural basal media (NBM) containing DMEM/F12 medium (Corning, 10-090-CV), supplemented with B27 (Gibco, 17504-044), epidermal growth factor (EGF) (20ng/ml, Sigma, E9644), basic fibroblast growth factor (bFGF) (20ng/ml, Sigma, F0291) and 1% antibiotic/antimycotic supplements (Corning, 30-004-CI). For cell passaging, GSCs were dissociated into single cells with Accutase (Sigma, A6964) once large neurospheres formed. U87 and 293T cells were obtained from American Type Culture Collection(ATCC) and grown in DMEM with 10% fetal bovine serum (FBS) (Sigma, F0926) and 1% antibiotic/antimycotic supplements (Corning, 30-004-CI).
Authentication	All the cells used in the study have been validated by STR DNA fingerprinting. The STR profiles were compared to online search database (DSMZ/ATCC/JCRB/RIKEN) of approximately 2500 known profiles; along with MD Anderson Cancer Center Characterized Cell Line Core (CCLC) database of approximately 2600 known profiles. The STR profiles of the cells used in the study were confirmed matching known DNA fingerprints.
Mycoplasma contamination	LookOut® Mycoplasma PCR Detection Kit(Sigma, MP0035) was used to confirmed the cells used were negative for mycoplasma contamination.
Commonly misidentified lines (See ICLAC register)	U87MG ATCC was used in the study as one additional GBM cell line. It is a GBM cell line that suitable for the addressing the scientific question in this study although it is not the original U87MG Uppsala cell line (https://www.cellosaurus.org/CVCL_0022). All the results obtained using this cell line were validated by patient-derived GSCs that were widely used in GBM filed.

Animals and other research organisms

Policy information about studies involving animals; ARRIVE guidelines recommended for reporting animal research, and Sex and Gender in Research

Laboratory animals	6-8 weeks old BALB/c Nude (nu/nu) were used in this study.
Wild animals	No wild animals were used in this study.
Reporting on sex	Female mice were used for GSC20 experiments, and equal numbers of male and female mice were initially used for GSC11 experiments (a small number were lost during guide-screw placement/implantation). Moreover, GSC20 and GSC11 were derived from male and female patients, respectively, therefore sex was not considered a biological variable.
Field-collected samples	No filed-collected samples were used in this study.
Ethics oversight	All animal studies were performed in accordance with NYU Langone Health Institutional Animal Care and Use Committee (IACUC) approved protocols.

Note that full information on the approval of the study protocol must also be provided in the manuscript.

Plants

Seed stocks	n/a
Novel plant genotypes	n/a
Authentication	n/a

Flow Cytometry

Plots

Confirm that:

- [x] The axis labels state the marker and fluorochrome used (e.g. CD4-FITC).
- [x] The axis scales are clearly visible. Include numbers along axes only for bottom left plot of group (a 'group' is an analysis of identical markers).
- [ ] All plots are contour plots with outliers or pseudocolor plots.
- [x] A numerical value for number of cells or percentage (with statistics) is provided.

Methodology

Sample preparation	Samples for cell cycle analysis and apoptosis analysis were prepared according to the manufacturers' manuals, Propidium Iodide Flow Cytometry Kit (Abcam, ab139418) for cell cycle analysis and FITC Annexin V apoptosis Detection Kit I (BD pharmingen, 556547) for apoptosis analysis. For HR/NHEJ reporter assay, cells were digested with accutase or 0.05% trypsin to single cells, washed and resuspended in 1× PBS, followed by filtering with Falcon Test Tube with Cell Strainer Snap Cap.For detecting cell surface sialylation, 5 x 105 GSC20 cells were suspended as single cells then washed with staining buffer (1 x PBS, 1mM CaCl2, 1mM MgCl2, 1% BSA) for three times. Cells were subsequently incubated with biotinylated Sambucus nigra agglutinin (SNA, 4 µg/ml, Vector Labs, B-1305-2), biotinylated Maackia amurensis Lectin II (MAL-II, 4 µg/ml, Vector Labs, B-1265-1), biotinylated peanut agglutinin (PNA, 4 µg/ml, Vector Labs, B-1075-5) or biotinylated Wheat germ agglutinin (WGA, 4 µg/ml, Vector Labs, B-1025-5) in staining buffer at 4 °C degree with rotation for 1 h. Cells stained with blank staining buffer without lectin were used as negative controls. The cells were then washed with cold staining buffer and then incubated with Streptavidin-conjugated Alexa Fluor 488 (2 µg/ml, Invitrogen, S11223) at 4 °C degree with rotation for 1 h. After washing with cold staining buffer three times, the fluorescence of the labelled cells was detected by flow cytometry (LSRII HTS, BD).
Instrument	BD LSR II HTS Flow Cytometer,FACSymphony™ A5 flow cytometer
Software	BD FACSDiva™ software, FlowJo v10 and ModFit LT.
Cell population abundance	Not applicable. This study did not involved cell sorting.
Gating strategy	Cells were first gated by FSC/SSC, followed by doublet discrimination gating . For cell cycle analysis, all the singlets were subjected to DNA content (PE channel intensity) analysis. For apoptosis analysis or HR/NHEJ reporter analysis, negtive and single-positive samples were used for compensation. To distinguish between positive and negative for each channel, negative samples were used to gate the boundaries. Example gating for HR/NHEJ experiment was shown in Supplementary Fig S7. For TNFR1 internalization assay, PI was used to exclude dead cells. Gating strategies are included Suplementary Fig. 16-19.

- [x] Tick this box to confirm that a figure exemplifying the gating strategy is provided in the Supplementary Information.
